# The association between obesity with treatment duration, ICU length of stay and the risk of death in critically ill patients with COVID‐19

**DOI:** 10.1002/edm2.458

**Published:** 2023-10-29

**Authors:** Maryam Gholamalizadeh, Mohammad Attari, Mahdi Mousavi, Soheila Shekari, Zahra Salimi, Asma Rajabi Harsini, Mobina Zeinolabedin, Amin Barzkar, Zahra Mahmoudi, Farkhondeh Alami, Samaneh Mirzaei Dahka, Somayeh Gholami, Masoume Rahvar, Masoume Pourtaleb, Sara Khoshdooz, Naser Kalantari, Saeid Doaei

**Affiliations:** ^1^ Student Research Committee, Cancer Research Center Shahid Beheshti University of Medical Sciences Tehran Iran; ^2^ Loghman Hakim Hospital Shahid Beheshti University of Medical Sciences Tehran Iran; ^3^ Student Research Center Tabriz University of Medical Sciences Tabriz Iran; ^4^ Department of Nutrition, Science and Research Branch Islamic Azad University Tehran Iran; ^5^ Nutrition and Metabolic Diseases Research Center Ahvaz Jundishapur University of Medical Sciences Ahvaz Iran; ^6^ Department of Clinical Nutrition School of Nutritional Sciences and Dietetics, Tehran University of Medical Sciences (TUMS) Tehran Iran; ^7^ Department of Community Nutrition School of Nutritional Sciences and Dietetics, Tehran University of Medical Sciences (TUMS) Tehran Iran; ^8^ Kerman University of Medical Sciences Kerman Iran; ^9^ Student Research Committee, Department of Nutrition, Faculty of Medicine Urmia University of Medical Sciences Urmia Iran; ^10^ School of Nursing and Midwifery Guilan University of Medical Sciences Rasht Iran; ^11^ Intensive Care Unit (ICU) Razi Hospital, Guilan University of Medical Sciences Rasht Iran; ^12^ School of Health Guilan University of Medical Sciences Rasht Iran; ^13^ Faculty of Medicine Guilan University of Medical Science Rasht Iran; ^14^ Department of Community Nutrition, Faculty of Nutrition and Food Technology, National Nutrition and Food Technology Research Institute Shahid Beheshti University of Medical Sciences Tehran Iran

**Keywords:** bodyweight, COVID‐19, ICU, mortality, obesity

## Abstract

**Background:**

Despite the confirmed association between higher BMI with increased risk of the acute respiratory distress syndrome (ARDS), the association between obesity with mortality in critically ill patients with coronavirus disease 2019 (COVID‐19) is not clear. The present study aimed to investigate the association between obesity with treatment duration, ICU length of stay, and the risk of death in critically ill patients with COVID‐19.

**Methods:**

This case–control study was performed on 223 patients with COVID‐19 including 148 surviving patients as the control group and 75 eventually dead patients as the case group in Rasht, Iran. Data on demographic factors, comorbidities, anthropometric measurements, the length of hospitalization and the mortality were obtained from patients' medical records.

**Results:**

The mortality rate was significantly associated with weight (OR = 1.04, 95% CI: 1.002–1.083, *p* = .04), but not with BMI after adjustments for age, gender, length of stay in ICU, chronic diseases and smoking. The results did not change after further adjustments for biochemical and pathological factors.

**Conclusions:**

Weight was positively associated with mortality after controlling for confounding variables. Further studies should consider the patient's body composition such as fat mass to establish the relationship between obesity and COVID‐19 outcomes.

## Graphical abstract

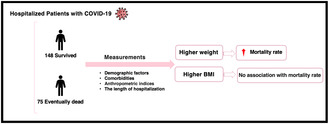

## 1 INTRODUCTION

Coronavirus disease 2019 (COVID‐19) is an infectious disease caused by severe acute respiratory syndrome coronavirus‐2 (SARS‐COV‐2)^1^ and more than 445 million cases and 6 million deaths have been reported worldwide.^2^ The last variant of COVID‐19, omicron, has been detected in more than 90 countries and 46 US states. Overall inpatient mortality was 13.2% and increasing to 55.9% in patients requiring mechanical ventilation.^3^ COVID‐19‐related complications which may lead to death included respiratory failure, acute respiratory syndrome (ARDS), sepsis and septic shock, acute renal injury, acute cardiac injury and multi‐organ failure.^4^ The risk factors of COVID‐19 reported by the World Health Organization (WHO) were age over 30 years, male gender, cardiovascular diseases, hypertension, diabetes, malignancy, and some environmental (e.g. low education, occupational risks) and lifestyle (e.g. obesity) factors.^5^, ^6^

Obesity, as a known risk factor for respiratory infections, was reported to be associated with unpleasant clinical outcomes in patients with COVID‐19.^7^, ^8^ A systematic review of the studies on obesity and mortality of COVID‐19 patients in the Asian and western countries reported that the mortality of COVID‐19‐hospitalized patients is related to obesity.^9^ The exact mechanism of the effect of obesity on patients with COVID‐19 is not yet clear. Obesity‐induced inflammation may play a crucial role in the immune system dysfunction in patients with COVID‐19.^10^ Some studies indicated that higher BMI is related to an increased risk of ARDS, hospital length of stay, and the need for invasive mechanical ventilation.^11^, ^12^

Several studies indicated obesity is a risk factor for critically ill and intubated patients.^10^, ^13^, ^14^, ^15^ One recent systematic review suggested that obesity is significantly associated with increased severity and higher mortality among COVID‐19 patients.^16^ On the other hand, contradictory results were reported on the association between obesity with ICU length of stay, clinical outcomes, and mortality of critically ill patients with COVID‐19. For example, in a recent study on older COVID‐19 patients, an inverse association between obesity and 30‐day mortality was observed even after adjusting for all already‐known markers of poor prognosis.^17^ Another study found that obesity is associated with increased risk for intubation or death from COVID‐19 in adults younger than 65 years, but not in adults aged 65 years or older.^18^

Obesity may influence the survival of critical ill patients with COVID‐19 through different mechanisms such as increased risk of systemic inflammatory response syndrome (SIRS).^19^ However, few retrospective studies were performed on the association of obesity with mortality in ICU patients with COVID‐19. So, this case–control study was conducted to investigate the association between obesity and the risk of mortality in critically ill patients with COVID‐19.

## 2 METHODS

### 2.1 Participants

This case–control study was conducted on 223 patients with COVID‐19, admitted to Razi Hospital from April to July 2019 in Rasht, Iran. The inclusion criteria were a confirmed diagnosis of COVID‐19 by a CT scan test and lung involvement, hospitalized in ICU for at least 24 h in a certain period (3 months from baseline). The exclusion criteria were age lower than 18 years, patients who died for other reasons except for COVID‐19, patients who did not have the required information in their medical files. Patients with COVID‐19 who died within 1 month of hospitalization were assigned to the case group (*n* = 75), and hospitalized patients who survived at least 1 month after admission to the hospital (recovered and discharged from the hospital) were assigned to the control group (*n* = 148). A 1:2 case to control ratio was used to calculate sample size with 95% CI, 80% power, and the odds ratio of a similar previous study^20^ using Open‐EPI online software.^21^

Data on demographic status (age, sex, height, and weight), co‐morbidities, pregnancy status and other personal information were obtained from the patient's medical files. The length of hospitalization and the mortality rate were investigated for all participants. The height (cm) and weight (kg) that were documented upon admission, either by self‐report or obtainment by nursing staff were used for BMI calculation as an indication of healthy bodyweight. The other clinical information of participants, including the report of lung CT scan and co‐morbidities including diabetes, respiratory diseases, transplantation and dialysis, were obtained from the patient's medical files. Also, in the control group who recovered and discharged, the required information was obtained from their medical files and, in the case of lack of knowledge, an oral interview was conducted in person or by telephone.

### 2.2 Statistical analyses

The independent *T*‐test and the chi‐squared test were used for comparison of the quantitative and qualitative variables between the groups, respectively. Different models of bi‐nominal logistic regression were used to investigate the association between COVID‐19 with weight and BMI and adjusting the effect of the confounding variables. The odds ratio (OR) and 95% CI were reported. All statistical analyses were SPSS statistical software (IBM SPSS Statistics 20; Chicago, IL, USA), and a *p*‐value <.05 was considered significant.

## 3 RESULTS

No difference was found regarding the mean age, sex, and the mean BMI between the case and control groups. The survived group had lower chronic diseases (24% vs. 44.6%, *p* = .003) and smoking (5.5% vs. 21.1%, *p* = .003) compared to the dead patients. There was no significant difference on the length of stay in the ICU between the case and control groups (Table 1).

**TABLE 1:** Characteristics of the participants.

	Survived patients	Median	Dead patients	Median	*p*
Age (years)	55.24 ± 15.45	55	58.49 ± 15.07	59	.14
Sex					
Males	71 (48%)		38 (50.7%)		.14
Females	77 (52%)		37 (49.3%)		
BMI (kg/m^2^)	26.69 ± 3.86		27.46 ± 4.46		.18
Weight (kg)	76.21 ± 7.22	75	77.00 ± 10.71	80	.68
Height (cm)	168.32 ± 14.32	170	78.23 ± 8.66	170	.37
Length of stay in ICU (days)	4.43 ± 1.37	4	4.57 ± 1.85	4	.50
Chronic diseases					
Yes	18 (24%)		66 (44.6%)		.003
No	57 (76%)		82 (55.4%)		
Smoking					
Yes	5 (5.5%)		32 (21.1%)		.003
No	70 (94.5%)		116 (78.9%)		

Abbreviations: APACHE II, The Acute Physiology and Chronic Health Evaluation; BMI, body mass index.

Regarding the biochemical and pathologic factors, the cases had abnormal levels of Be (1.57 ± 5.02 vs. −5.70 ± 7.40 mEq/litre), SGPT (30.75 ± 32.24 vs. 80.71 ± 171.34 1 U/L), SGOT (39.56 ± 28.09 U/L vs. 87.58 ± 165.53 U/L), the MAP (71.86 ± 12.16 mm Hg vs. 59.61 ± 6.88 mm Hg), PCO_2_ (43.17 ± 8/92 vs. 49.85 ± 15.20), HCO_3_ level (25.72 ± 4.81 mEq vs. 20.82 ± 12.15 mEq), PTT (33.57 ± 11.43 vs. 40.31 ± 16.55 s), GCS (14.34 ± 1.98 vs. 7.83 ± 1.08) compared to the controls (All *p* < .05) (Table 2).

**TABLE 2:** Biochemical and pathological factors among the case and control groups at baseline.

	Survived patients	Dead patients	Normal range	*p*
BS (mg/dL)	147.88 ± 55.11	151.15 ± 54.52	<140	.675
Na (mmol/L)	138.55 ± 15.51	140.23 ± 19.28	135–145	.484
K (mEq/L)	3.83 ± 0.50	4.11 ± 0.81	3.6–5.2	.002
BUN (mg/dL)	29.57 ± 24.82	41.79 ± 29.38	6–24	.001
Cr (mg/dL)	2.80 ± 9.94	2.83 ± 7.63	0.7–1.3 for men 0.6–1.1 for women	.980
Alb (g/dL)	2.82 ± 0.48	2.71 ± 0.60	3.5–5.4	.141
HCT (%)	32.17 ± 6.74	31.25 ± 6.38	41–50 for men 36–48 for women	.329
Ca (mg/dL)	8.35 ± 1.22	7.85 ± 0.87	8.6–10.3	.003
P (mg/dL)	5.16 ± 0.83	4.88 ± 1.50	2.5–4.5	.122
MAP (mm Hg)	71.86 ± 12.16	59.61 ± 6.88	70–100	<.001
Band cell (%)	6.58 ± 4.37	6.00 ± 3.09	3–5	.692
O_2_Sat (%)	87.04 ± 11.07	84.41 ± 13.46	95–100	.123
PH	7.34 ± 0.34	7.25 ± 0.10	7.35–7.45	.031
PO_2_ (mm Hg)	78.10 ± 53.71	78.34 ± 46.55	75–100	.973
PCO_2_ (mm Hg)	43.17 ± 8.92	49.86 ± 15.20	35–45	<.001
HCO_3_ (mEq)	25.72 ± 4.81	20.82 ± 12.15	22–26	<.001
BE (mEq/L)	1.57 ± 5.02	−5.70 ± 7.40	−2 to +2	<.001
CRP (mg/dL)	11.52 ± 56.45	12.37 ± 60.94	<3	.410
WBC (10^3^/μL)	11.46 ± 4.66	12.19 ± 5.50	4500–11,000	.301
ALK.PH (U/L)	197.72 ± 60.68	233.23 ± 151.38	44–147	.018
PT (s)	13.93 ± 1.96	15.48 ± 3.97	11–13.5	<.001
PTT (s)	33.57 ± 11.43	40.31 ± 16.55	25–35	<.001
INR	1.51 ± 1.49	2.52 ± 8.90	0.8–1.1	.185
ESR (mm/h)	69.25 ± 37.22	93.26 ± 133.93	0–15 in men 0–20 in women	.241
Urine volume (mL/day)	3543.07 ± 1837.75	2196.81 ± 1191.73	800–2000	<.001
SGPT (U/L)	30.75 ± 32.24	80.71 ± 171.34	7–56	.001
SGOT (U/L)	39.56 ± 28.09	87.58 ± 165.53	8–45	.001
TBIL (mg/dL)	1.21 ± 1.48	1.53 ± 1.66	1.2	.176
DBIL (mg/dL)	0.41 ± 0.58	0.61 ± 0.97	0.3	.078
Neutrophils (%)	78.32 ± 7.90	81.17 ± 8.22	40–60	.013
Lymphocytes (%)	13.01 ± 6.69	11.09 ± 5.32	20–40	.033
Monocytes (%)	4.92 ± 2.23	4.33 ± 2.33	2–8	.070
Eosinophils (%)	3.60 ± 3.38	2.85 ± 3.28	1–4	.135
GCS	14.34 ± 1.98	7.83 ± 1.08	<8	<.001
APACH II	30.17 ± 5.52	32.92 ± 4.09	23	<.01
Hb (g/dL)	10.25 ± 1.97	9.63 ± 2.14	14–18 in men 12–16 in women	.034
Plt (10^3^/μL)	208.98 ± 107.98	184.65 ± 124.93	150–450	.134

Abbreviations: Alb, albumin; ALK, alkaline phosphatase; BS, blood sugar; BUN, blood urea nitrogen; CR, creatinine; CRP, c‐reactive protein; DBIL, direct bilirubin; ESR, erythrocyte sedimentation rate; GCS, Glasgow Coma Scale; Hb, Haemoglobin; HCT, haematocrit; INR, international normalized ratio; MAP, microtubule‐associated protein; Plt, platelet; PT, Prothrombin time; PTT, partial thromboplastin time; SGOT, Aspartate transaminase; SGPT, Alanine transaminase; TBIL, total bilirubin.

As shown in Table 3, there was a significant association between the mortality rate and weight after adjustment for age, gender, length of stay in ICU, chronic diseases and smoking (OR = 1.04, 95% CI: 1.002–1.083, *p* = .04) (Model 1). The result remained significant after additional adjustments for biochemical and pathological factors (OR = 1.041, 95% CI: 1.001–1.083, *p* = .046) (Model 2). Regarding the association between mortality and obesity, there was no association between the mortality rate with BMI after adjustment for age, gender, length of stay in ICU, chronic diseases and smoking (OR = 1.067, 95% CI: 0.995–1.144, *p* = .069) (Model 1) and after additional adjustments for biochemical and pathological factors (OR = 1.056, 95% CI: 0.977–1.141, *p* = .173) (Model 2).

**TABLE 3:** The association between weight and BMI with mortality.

	Model 1		Model 2	
	OR (95% CI)	*p*	OR (95% CI)	*p*
Weight	1.04 (1.002–1.083)	.040	1.041 (1.001–1.083)	.046
BMI	1.067 (0.995–1.144)	.069	1.056 (0.977–1.141)	.173

*Note*: Model 1: Adjusted for age and gender, length of stay in ICU, chronic diseases, and smoking. Model 2: Further adjustments for biochemical and pathological factors.

## 4 DISCUSSION

The present study found a positive association between body weight and mortality in patients with COVID‐19. No significant association was found between BMI and the risk of death in critically ill patients with COVID‐19. Few studies were performed on the association between obesity and clinical outcomes of critically ill patients with COVID‐19. In line with the results of the present study, some previous studies showed that body weight was associated with unpleasant complications in patients with COVID‐19.^22^ Furthermore, some other studies reported that obesity is a risk factor for critically ill and intubated patients.^13^, ^14^ Gong et al. and Bajwa et al. found a significant relationship between higher BMI and the duration of hospitalization and increased risk of ADRS.^11^

In contrast with the results of the present study, several studies reported that higher BMI is positively associated with higher risk of COVID‐19 complications.^23^, ^24^ However, the association between obesity and COVD‐19 may be influenced by some other factors such as age and gender. For example, Tartof et al. reported that obesity was associated with the risk of mortality in patients with COVID‐19 only in male patients younger than 60 years.^25^ Interestingly, the molecular mechanisms of COVID‐19 are reported to be varied in different ages and genders.^26^ It is possible that the contradictory results on the association between obesity and COVID‐19 may be related to differences in the pathogens of COVID‐19 in different ages and genders. Moreover, using BMI in clinical investigations may be misleading in some cases. For example, people with higher muscle mass such as athletes have higher BMI compared with the others. The single use of BMI for the obesity assessment is reported to be misleading and should be used in conjunction with other obesity indices.^27^

The exact mechanism of the effect of bodyweight on COVID‐19 is not yet clear. Several studies that have suggest various mechanisms.^28^, ^29^ One proposed theory is that obesity is associated with chronic low‐grade inflammation and immune dysregulation.^29^ The increased amounts of cytokines such as TNFα, IL‐1, IL‐6 and monocyte chemoattractant protein (MCP‐1) may lead to defective function of innate and adaptive immunity. Metabolic complications and the chronic imbalance in the hormonal and adipocytokine microenvironment are reported to be major determinants in the severity of viral infections in obesity.^30^ In obese persons, there are reduced macrophage activation, increased proinflammatory cytokine production, and impaired B and T cell activation.^31^ Another proposed mechanism is the overexpression of angiotensinogen (AGT) and over‐activation of the renin‐angiotensin‐aldosterone system (RAS) system in adipose tissue resulting in an increased production of angiotensin II.^32^ In the present study, body weight regardless of height and BMI was related to death in patients with COVID‐19. It is possible that an increase in the amount of body adipose tissue, regardless of whether it is associated with obesity or not, is associated with an increased risk of death in these patients due to a change in the level of secretion of some hormones and the level of resistance to some adipose‐related factors.^30^

Further studies are required considering the limitations of this study, including the small size of the sample, the different medical backgrounds of the patients, to confirm these findings and to identify the underlying mechanisms of the effects of bodyweight on COVID‐19.

## 5 CONCLUSION

The results from this case–control study indicate that higher weigh, not BMI, is associated with higher mortality in critically ill patients with COVID‐19 after controlling for confounding variables. Weight loss may reduce COVID‐19 severity and hospitalization. These findings need to be confirmed by future large‐scale studies that include patients of various pathological backgrounds. In addition, further studies should consider the patient's body composition, such as fat mass, to establish the relationship between obesity and COVID‐19 outcomes. If the results of this study are confirmed in future research, weight loss diets in obese or overweight people can be recommended as a strategy to prevent the exacerbation of the symptoms of COVID‐19.

## AUTHOR CONTRIBUTIONS

**Mahdi Mousavi:** Software (equal). **Zahra Salimi:** Validation (equal). **Asma Rajabi:** Investigation (equal). **Mobina Zeinolabedin:** Formal analysis (equal). **Amin Barzkar:** Validation (equal). **Zahra Mahmoudi:** Validation (equal). **Farkhondeh Alami:** Validation (equal). **Samaneh Mirzaei Dahka:** Methodology (equal). **Somayeh Gholami:** Writing – original draft (equal). **Masoume Rahvar:** Software (equal). **Masoume Pourtaleb:** Methodology (equal). **Sara Khoshdooz:** Visualization (equal). **Maryam Gholamalizadeh:** Formal analysis (equal). **Mohammad Attari:** Methodology (equal). **Saeid Doaei:** Formal analysis (equal). **Soheila Shekari:** Validation (equal). **Naser Kalantari:** Methodology (equal).

## FUNDING INFORMATION

This study is financially supported by the Student Research Committee of Shahid Beheshti University of Medical Sciences, Tehran, Iran (Code 1399/63396). Thanks to all the colleagues in the research committee for their nice cooperation.

## CONFLICT OF INTEREST STATEMENT

The authors declare no competing interests.

## ETHICS STATEMENT

The protocol of this study was approved by the ethics committee of Shahid Beheshti University of Medical Sciences, Tehran, Iran (Code: IR.SBMU.RETECH.REC.1399.1057).

## CONSENT TO PARTICIPATE

Written consent was obtained from the participants or their first degree relatives (for deceased patients) to participate in the study.

## Data Availability

Data is available upon on request of corresponding author.
